# Comprehensive analysis of early fractional anisotropy changes in acute ischemic stroke

**DOI:** 10.1371/journal.pone.0188318

**Published:** 2017-11-30

**Authors:** Anna Christina Alegiani, Simon MacLean, Hanna Braass, Susanne Siemonsen, Christian Gerloff, Jens Fiehler, Tae-Hee Cho, Laurent Derex, Marc Hermier, Yves Berthezene, Norbert Nighoghossian, Götz Thomalla

**Affiliations:** 1 Department of Neurology, University Medical Center Hamburg-Eppendorf, Hamburg, Germany; 2 Department of Neuroradiology, University Medical Center Hamburg-Eppendorf, Hamburg, Germany; 3 Department of Stroke Medicine, Université Lyon, Lyon, France; 4 Department of Neuroradiology, Université Lyon, Lyon, France; University of Münster, GERMANY

## Abstract

**Background and purpose:**

Cerebral ischemia leads to a rapid decrease of the apparent diffusion coefficient. For fractional anisotropy both increase and decrease have been reported in acute ischemic stroke. Aim of this study was to characterize early water diffusion changes in a homogenous group of acute stroke patients and to clarify the issue of early fractional anisotropy changes and their relation to time from symptom onset.

**Methods:**

MRI data of patients with acute ischemic stroke examined by diffusion tensor imaging within 8h after symptom were analyzed. We calculated fractional anisotropy, eigenvalues and the isotropic and anisotropic components of the diffusion tensor. The values were calculated as ratios between the ischemic lesion and a mirror region in the unaffected side and correlated with clinical parameters.

**Results:**

We included 63 patients: 49% female, mean age 69 ± 14 years, median NIHSS on admission 9 (IQR 4–14). For the whole sample, mean fractional anisotropy was increased (ratio: 1.083 ± 0.168), while all other diffusion parameters were decreased. Both the isotropic and anisotropic component of the diffusion tensor were decreased with a more pronounced decrease of the isotropic component (ratios: isotropic = 0.730 ± 0.106, anisotropic = 0.788 ± 0.127; p<0.001). There was no correlation of fractional anisotropy with time from symptom onset. Looking at individual patients, fractional anisotropy was increased in 70%. There were no differences in clinical characteristics between patients with increased and decreased fractional anisotropy.

**Conclusion:**

Fractional anisotropy increase in acute stroke results from a more pronounced decrease of the isotropic diffusion component and is not related to time from symptom onset. Thus, fractional anisotropy is not helpful as a surrogate marker of lesion age in the very first hours of stroke.

## Introduction

Acute cerebral ischemia leads to changes in water diffusion that can be captured by diffusion weighted imaging (DWI). Most prominent is the rapid decrease of the apparent diffusion coefficient (ADC) which enables the identification of acute ischemic stroke lesions with a high sensitivity [1]. The use of DWI is well established in the routine diagnostic work-up of stroke patients, but there are limitations to the use of DWI. To a certain extent acute DWI lesions may be reversible [2], and DWI does not allow for an estimation of lesion age within the first 24 hours of stroke [3].

Diffusion tensor imaging (DTI) applying DWI along ≥6 directions allows for a more comprehensive characterization of diffusion characteristics of brain tissue [4]. Different parameters may be calculated to describe tissue diffusion properties, including the diffusivity along the principal axes of the diffusion tensor, i.e. the three eigenvalues (ʎ1–3), as well as fractional anisotropy (FA), the most commonly used measure of diffusion anisotropy.

In acute ischemic stroke, both increased and decreased FA has been reported [5,6,7], whereas FA is always decreased in the subacute to chronic stage of cerebral ischemia [8,9]. While the decrease of FA during the course of ischemia reflects irreversible tissue damage it is not clear whether increased FA in the first hours of stroke reflects meaningful physiological characteristics of brain tissue [10]. In imaging studies of stroke patients within the first 12 hours to days of stroke onset a certain relation of FA to time from symptom onset was observed. Thus, it was suggested that FA might help estimate lesion age in patients with unknown time of symptom onset [7,11,12]. A contradicting report explained increased FA values as a result of ratio-metric calculation of FA, i.e. changes in the ratio between the anisotropic and the isotropic components of the diffusion tensor in acute ischemia [13].

The aim of our study was to comprehensively characterize early water diffusion changes in a larger homogenous group of acute stroke patients in order to clarify the issue of early FA changes and their possible relation to clinical parameters like time from symptom onset.

## Materials and methods

We analyzed MRI data of patients with acute ischemic stroke studied by MRI including DTI within 8 hours of symptom onset in two stroke centers (Hospices Civils de Lyon and University Medical Center Hamburg-Eppendorf) between October 2008 and September 2009. Part of the sample represents a subgroup of the I-KNOW database [14]. Inclusion criteria were acute ischemic stroke and MRI including DWI within 8 hours of symptom onset. The study was approved by the Ethics Hamburg chamber of physicians (No 2666). Patients provided informed written consent to have data from their medical records used in research. Reporting recommendations following the STROBE Statement were incorporated [15]. 72 patient records were available, but 9 had to be excluded due to contralateral infarcts, missing onset time or infarct size.

### MRI protocol

Magnetic resonance images were acquired on 1.5 clinical whole-body units (Magnetom Symphony/Sonata; Siemens, Erlangen, Germany). The protocol included a single shot spin-echo echo-planar DTI sequence with diffusion weighting along 12 directions, field of view = 24x24 cm, matrix 128x128, slice thickness 3 mm, b-value 1000 s/mm^2^, TR >5000 s. The protocol also included a conventional single shot spin-echo echo-planar DWI sequence with a TR/TE of 2600/77, slice thickness of 5 mm, 1.5 mm gap, applying three *b* values (0, 500, and 1000 s/mm^2^).

### Postprocessing

DTI data were processed using FSL (FMRIB Software Library, Centre for Functional MRI of the Brain, University of Oxford). The diffusion tensor (D) for each voxel was calculated and maps of FA, mean diffusivity (MD), and eigenvalues were calculated. In addition we calculated the isotropic (p) and anisotropic (q) components of the diffusion tensor, where p is identical to MD and q is calculated by

$$q=\sqrt{(\lambda_{1}-D)²+(\lambda_{2}-D)²+(\lambda_{3}-D)²}$$

[13]. FA is calculated by

$$FA=\sqrt{\frac{3}{2}}\frac{\sqrt{(\lambda_{1}-D)²+(\lambda_{2}-D)²+(\lambda_{3}-D)²}}{\sqrt{\lambda_{1}^{2}+\lambda_{2}^{2}+\lambda_{3}^{2}}}$$

*or including q*:

$$FA=\sqrt{\frac{3}{2}}\frac{q}{\sqrt{\lambda_{1}^{2}+\lambda_{2}^{2}+\lambda_{3}^{2}}}$$

All images were registered to MNI space. For quantitative analysis, CSF was automatically excluded from the segmented brain tissue on diffusion weighted (b = 1000) images using an upper threshold of 1200 × 10–6 mm^2^/s [16].

### Volume of interest analysis—Acute DWI lesion and mirror lesion

**Fig 1:**
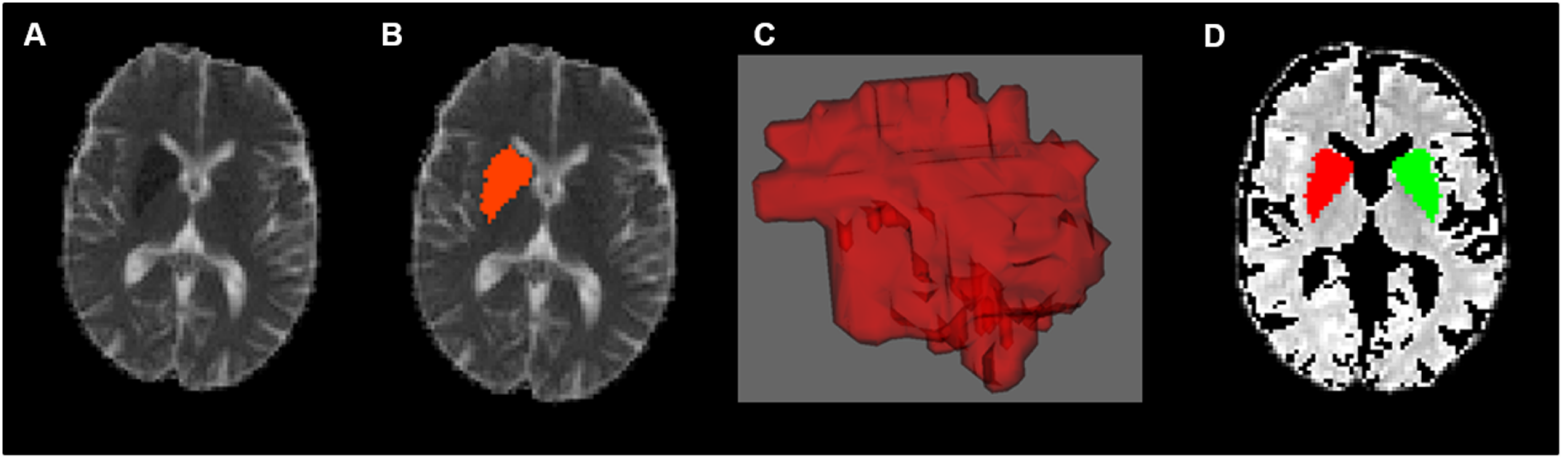
Volume of interest definition. Illustration of the Infarct region (region of interest) in the diffusion weighted image, the manually defined mask (using Create Mask, FSL), resulting 3D mask and the mirrored region after exclusion of CSF.

Volumes of interest (VOI) of the acute DWI lesion were manually defined on a voxel-by-voxel basis based on ADC maps with Create Mask (FSL) (for an example see Fig 1). Minimal VOI size was 4 voxels on more than one slice. DWI lesion VOI was mirrored along the x-axis in order to obtain mirror VOI from the unaffected hemisphere for comparison. Values for each VOI were obtained by averaging all voxel within the VOI. FA, MD/p, q and eigenvalues were compared for the lesion VOI and mirror VOI and affected and unaffected side. For all diffusion parameters ratios between the stroke lesion VOI and the mirror VOI were calculated. Contralateral stroke or severe leukoaraiosis were excluded before using the contralateral VOI.

### Clinical parameters

We surveyed demographic data (age and gender), time from symptom onset, severity of symptoms on admission assessed by the National Institutes of Health Stroke Scale (NIHSS), blood pressure and selected laboratory values that might potentially influence diffusion characteristics (e.g. blood glucose, blood counts).

### Statistical analysis

We used SPSS (21.0.0.0., SPSS Inc., Chicago, IL) for statistical analysis. Values are presented as mean ± SD or median and interquartile range. Group comparison of diffusion parameters between hemispheres (affected vs. unaffected) was made using the related Samples Wilcoxon Signed Rank Test, and between patient groups (patients with increased FA vs. patients with decreased FA) using the Mann-Whitney U Test. Pearson correlation coefficient was calculated to test for correlation of ratios of p and q, of p and q with FA, and between diffusion parameters and clinical characteristics. Ratios of p and q were compared using the related Samples Wilcoxon Signed Rank Test.

## Results

Of 63 patients studied, 31 (49%) were female, mean age was 69 years. Median time from symptom onset to admission was 129min, median NIHSS on admission was 9 (for detailed information on clinical characteristics see Table 1).

**Table 1:** Demographic, clinical features and imaging data.

	Total (n = 63)
gender female, n (%)	31 (49.2)
age, mean ± SD [years]	69 ± 14
time stroke to mri, median (IQR, min)	129 (146–166)
NIHSS, median IQR	9 (4–14)
SBP, mean ± SD [mmHg], n = 62*	152 ± 27
blood glucose, mean ± SD [mg/dl], n = 60*	124 ± 35
hematocrit, mean ± SD [%], n = 53*	39 ± 6
leucocyte count, mean ± SD [*10^9^/l]	8.9 ± 3.4
plateled count, mean ± SD [*10^9^/l]	269 ± 101
side of infarction, left n (%)	36 (57.1)
lesion size, mean ± SD [ml]	19.45 ± 28.30
infarct classification	
lacunar infarction, n (%)	13 (20.6)
< 1/3 of media territory, n (%)	28 (44.4)
1/3 to 2/3 of media territory, n (%)	12 (19.0)
> 2/3 of media territory, n (%)	10 (159)

* in case of missing data number of patients with data available are given;

NIHSS = National Institute of Health Stroke Scale; SBP = systolic blood pressure; SD = standard deviation; IQR = interquartile range

Across the whole group, all diffusion parameters were significantly different between lesion VOI and the mirror VOI in the unaffected hemisphere. Mean FA values in the ischemic lesion were increased (0.313 vs. 0.292, p <.001) with a mean FA ratio of 1.083. This resulted from an increased FA in 44/63 patients (69.8%), while FA was decreased in 19/63 patients (30.2%). All other diffusion parameters were decreased in virtually all patients with ratios ranging between 0.708 for the 3^rd^ eigenvalue and 0.788 for the anisotropic component of the diffusion tensor (q) (see Table 2).

**Table 2:** Values of FA, MD, eigenvalues (λ1, λ2 and λ3) and q divided into affected and unaffected side.

	affected side, as	unaffected side, us	group comparison	ratio
	mean ± SD	mean ± SD	p value**	mean ± SD
*FA*	0.313 ± 0.086	0.292 ± 0.080	<.001	1.083 ± 0.168
MD = p*	0.591∙10^−3^ ± 0.094∙10^−3^	0.814∙10^−3^ ± 0.086∙10^−3^	<.001	0.730 ± 0.106
λ1*	0.784∙10^−3^ ± 0.114∙10^−3^	1.062∙10^−3^ ± 0.100∙10^−3^	<.001	0.741 ± 0.102
λ2*	0.564∙10^−3^ ± 0.097∙10^−3^	0.773∙10^−3^ ± 0.100∙10^−3^	<.001	0.733 ± 0.104
λ3*	0.428∙10^−3^ ± 0.101∙10^−3^	0.607∙10^−3^ ± 0.101∙10^−3^	<.001	0.708 ± 0.120
q*	0.267∙10^−3^ ± 0.075∙10^−3^	0.343∙10^−3^ ± 0.087∙10^−3^	<.001	0.788 ± 0.127

FA = fractional anisotropy; λ1 = first eigenvalue; λ2 = second eigenvalue; λ3 = third eigenvalue; MD (p) = mean diffusivity, q = anisotropic component (according to Green et al. 2002^13^); as = affected side; us = unaffected side; all values mean ± standard deviation (SD);

* absolute values in mm^2^/s;

**related-Samples Wilcoxon Signed Rank Test

**Fig 2:**
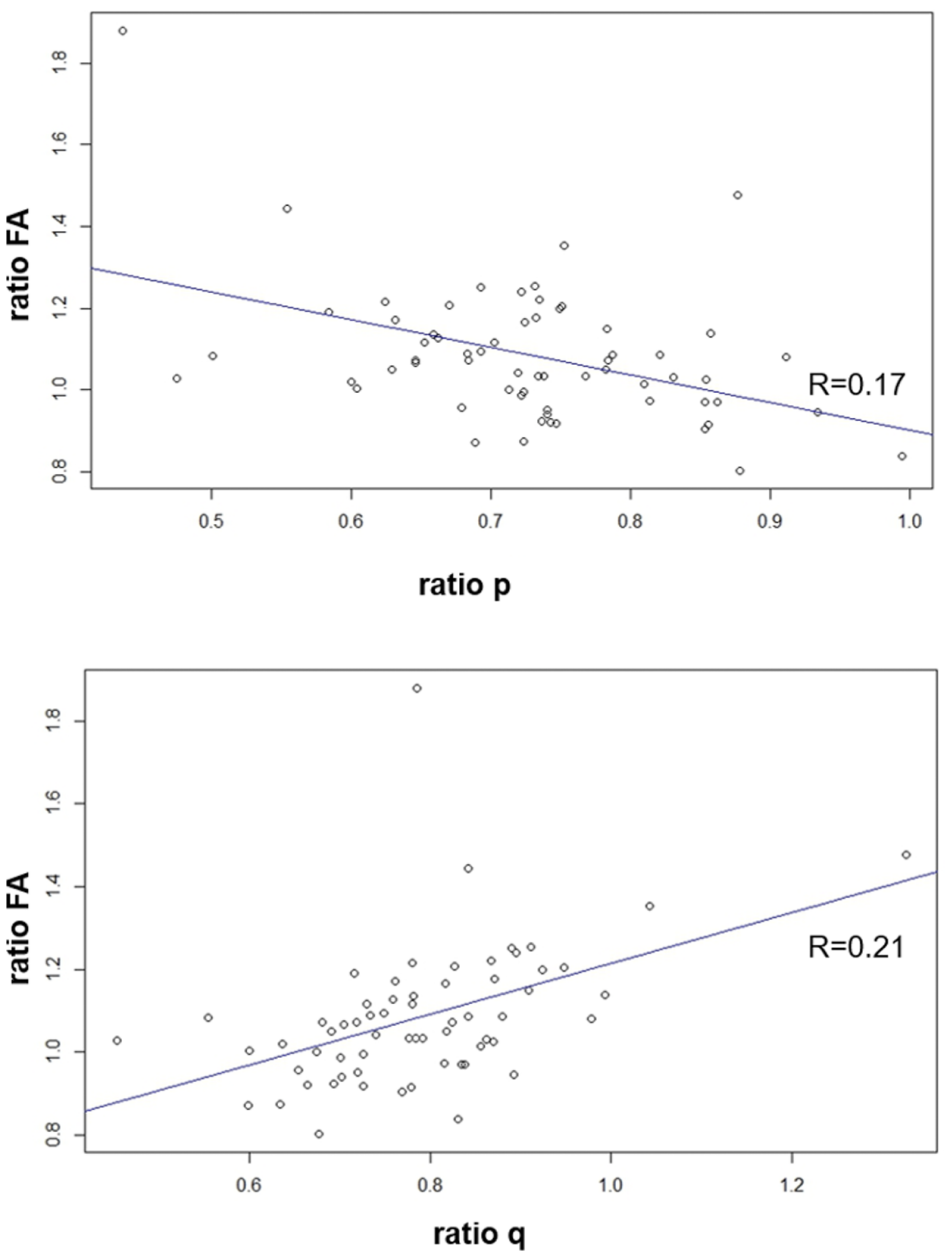
Ratio of FA versus p and q. The ratio of FA is plotted against p and q, showing FA is increasing with increasing q.

If the ratio of FA is plotted against p and q, FA is increasing with increasing q (see Fig 2).

**Fig 3:**
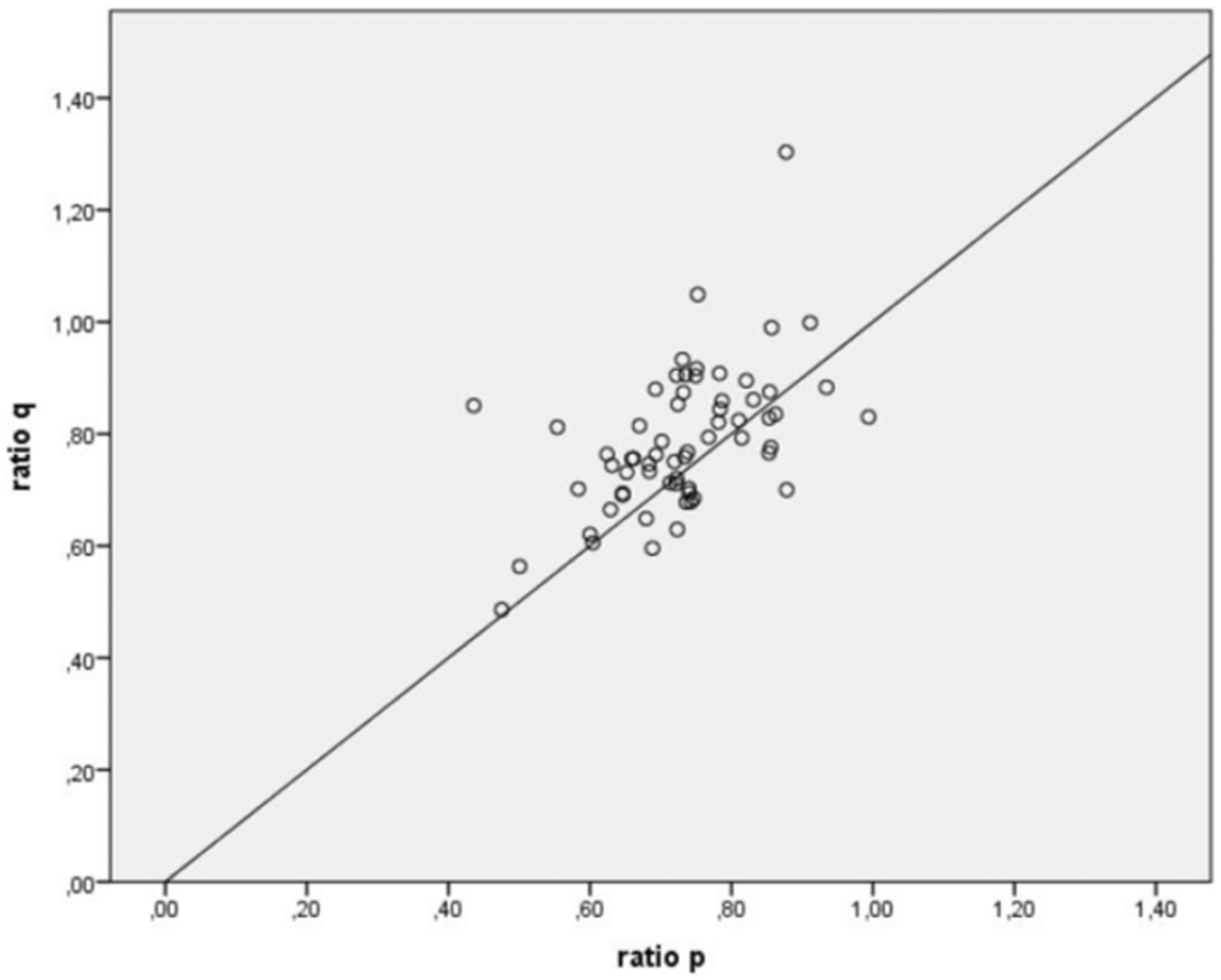
Ratios of isotropic components (p) versus anisotropic components (q) of the diffusion tensor. The ratios of isotropic components (p) are plotted against the anisotropic components (q) of the diffusion tensor.

Ratios of the isotropic (p) and anisotropic (q) components of the diffusion tensor showed a similar gradient (R^2^ = 0.28, p<0.001; see Fig 3), but with a significantly more pronounced reduction of p (mean ratios p = 0.730, q = 0.788; p<0.001).

**Fig 4:**
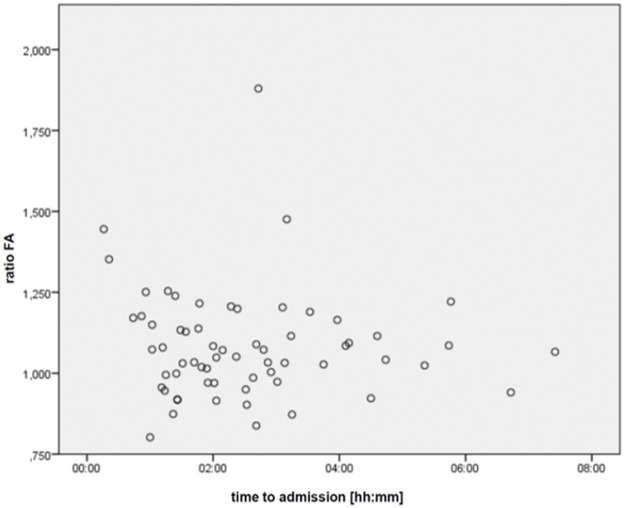
Ratio of FA versus time to MRI. The ratio of FA is plotted against the time to MRI.

The FA ratio showed no significant correlation to time from symptom onset (R = -0.072, p = .576) (see Fig 4).

To further elucidate factors influencing the direction of acute FA changes, we compared imaging and clinical parameters between patients with increased FA (rFA>1) and those with decreased FA (rFA <1). Groups were comparable for demographic parameters, clinical features and tested laboratory values, while absolute values of MD, ʎ2 and ʎ3 were smaller in patients with increased FA (see Table 3).

**Table 3:** Group comparison according to early FA increase or decrease.

	FA ratio >1 (n = 44)	FA ratio <1 (n = 19)	p-value*
gender, female, n (%)	22 (50.0)	9 (47.4)	.259
age, mean±SD [years]	68 ± 15	70 ± 13	.627
time stroke to MRI, median (IQR min)	139 (89–207)	121 (82–161)	.410
NIHSS, median (IQR)	9 (5–16)	8 (4–17)	.070
RR systolic, mean±SD [mmHg]	153 ± 29	148 ± 23	.240
blood glucose, mean±SD [mg/dl]	126 ± 34	120 ± 36	.008
hematocrit, mean±SD [%]	38.6 ± 5.9	40.3 ± 4.5	.369
Leucocyte count, mean±SD [*109/l]	9.0 ± 3.4	8.7 ± 3.6	.659
Plateled count, mean±SD [*109/l]	273 ± 101	260 ± 102	.389
lesion size, mean±SD [ml]	18.87 ± 28.52	20.82 ± 28.49	.695
lacunar infarction, n (%)	10 (22.7)	3 (15.8)	.883
< 1/3 of media territory, n (%)	18 (40.9)	10 (52.6)	
1/3 to 2/3 of media territory, n (%)	9 (20.5)	3 (15.8)	
> 2/3 of media territory, n (%)	7 (15.9)	3 (15.8)	
MD (p), mean±SD [*10–3 mm2/s]	0.568∙10–3 ± 0.072	0.646∙10–3 ± 0,117	.008
λ1, mean±SD [*10–3 mm2/s]	0.767∙10–3 ± 0.108	0.824∙10–3 ± 0.121	.119
λ2, mean±SD [*10–3 mm2/s]	0.539∙10–3 ± 0.071	0.620∙10–3 ± 0.124	.004
λ3, mean±SD [*10–3 mm2/s]	0.400∙10–3 ± 0.073	0.494 10–3 ± 0.126	.001
q, mean±SD [*10–3 mm2/s]	0.277∙10–3 ± 0.078	0.244∙10–3 ± 0.064	.005

MD (p) = mean diffusivity, q = anisotropic component (referred to Green et al. 2002); λ1 = first eigenvalue; λ2 = second eigenvalue; λ3 = third eigenvalue;

*Mann-Whitney U Test

## Discussion

We studied FA and eigenvalues of diffusion tensor imaging in 63 acute stroke patients within 8 hours after symptom onset. As a main result, FA was increased in the ischemic lesion as compared to the unaffected hemisphere in about two third of patients resulting in an increase of the mean FA ration for the whole sample. As expected, all other diffusion parameters including mean diffusivity and the eigenvalues showed a clear reduction in the ischemic lesion. Both the isotropic and anisotropic component of the diffusion tensor were decreased with a more pronounced decrease of the isotropic component. In line with previous findings of Green et al. [13], showing elevation of FA when there is a larger reduction of the isotropic mean diffusivity, i.e. p, than the anisotropic component of diffusion, i.e. q. In this context the significant stronger decrease of q indicates that FA changes are stronger driven by q changes than p changes. According to the formula for FA, a higher value for q results in a higher value of FA. This finding explains the increase of FA in our sample reflecting the altered ratio between anisotropic and isotropic diffusion components.

Thus, we conclude that acute FA changes were related to individual components of tissue water diffusion but not to clinical parameters such as time from symptom onset.

Our findings of increased FA in cerebral ischemia within the first hours of stroke are in line with previous reports [5,6,7]. There might be uncertainty that decreased FA reflects a reduced degree of water diffusion restriction along a certain direction, but not necessarily irreversible tissue damage. Increased FA, however, is not a uniform pattern in acute stroke, as there are also patients with rather unchanged or even decreased FA in the ischemic lesion both in our data as well as in previous reports [7]. Resulting from these observations, the question was raised whether increased FA in the first hours of stroke reflects meaningful physiological characteristics of brain tissue and allows infer on clinical characteristics such as lesion age [10]. In contrast to this assumption, a previous small study of 10 patients studied within 27 hours of stroke onset analyzed alterations of the anisotropic and isotropic components of the diffusion tensor within the ischemic lesion and attributed increased FA values to the ratio-metric calculation of FA, i.e. to a change in the ratio between the anisotropic and the isotropic components of the diffusion tensor [13].

Our results confirm these findings in a much larger sample studied within the first 8 hours of symptom: water diffusion was reduced along all directions of the diffusion tensor, but the isotropic components of the tensor showed a stronger reduction than the anisotropic components, resulting in an increased ratio of anisotropic/isotropic components and thus an increased FA. Group comparison of patients with increased and decreased FA further suggests that the imbalance between reduction the isotropic and anisotropic diffusion components relates to the severity of diffusion changes. Absolute values of mean diffusivity and of the smaller eigenvalues were smaller in Patients with FA ratio >1.

**Fig 5:**
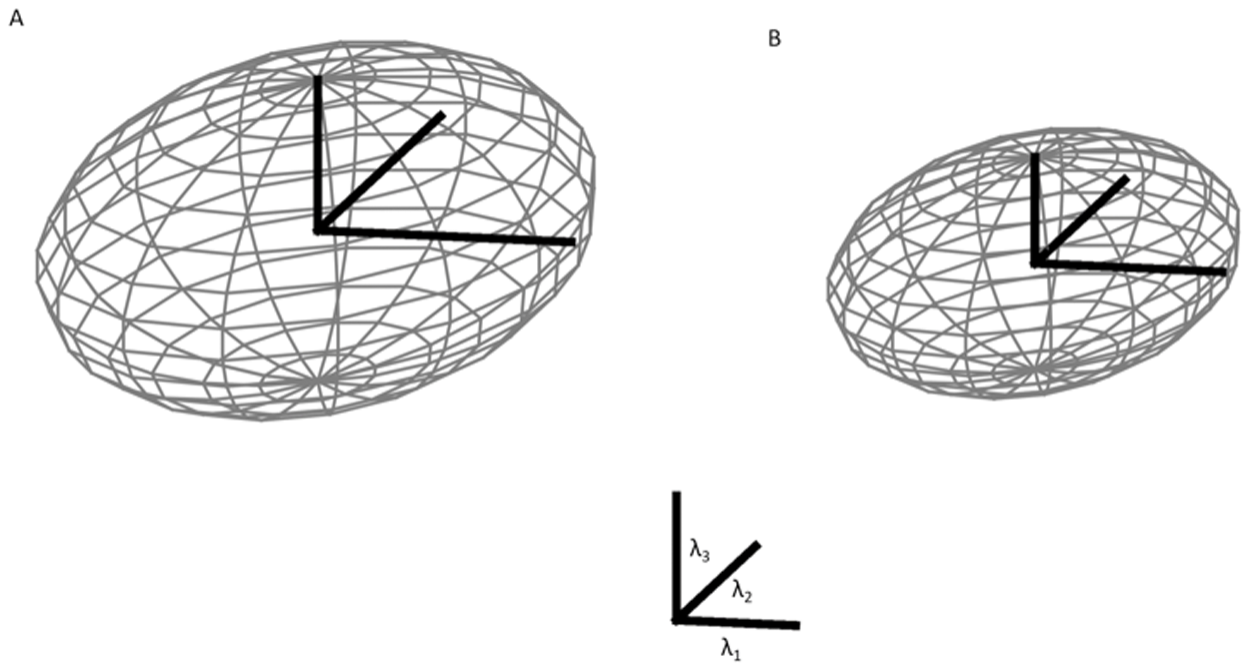
Mapping of the eigenvalues as ellipsoids, unaffected side and affected side. Mapping of the eigenvalues as ellipsoids divided into unaffected side (A) with FA = 0.292 and affected side (B) with FA = 0.313.

Fig 5 visualizes the mean diffusion tensor and illustrates the changes of water diffusion in the ischemic lesion as compared to contra lateral healthy tissue. Overall, the ellipsoid describing the diffusion tensor in the ischemic region is smaller with reduced diffusivity along all principal axes (eigenvalues) as compared to healthy tissue. However, as the reduction of diffusion is more pronounced for the smaller eigenvalues (ʎ2 and 3) than for the first eigenvalue (ʎ1), the tensor though overall smaller gets slightly more “cigar-like” shape, reflected by a slightly increased FA.

We did not observe any association of acute FA changes with clinical parameters. Most importantly, there was no correlation of rFA with time from symptom onset. This in contrast to previous reports that observed a correlation of acute FA changes with time from symptom onset [12]. The different results in our study may result from the more narrow time window in our sample (<8 hours of symptom onset) with a mean time from symptom onset to MRI of about 2.5 hours, thus reflecting the time period in which the most relevant treatment decisions need to be made. Previous studies included patients up to several days after symptom onset [17]. Given the well described decrease of FA in the later acute and subacute time window [8], it does not come as a surprise that mixing patients in the acute and early subacute stage in the analysis of FA changes may well identify a relation of FA changes to time from symptom onset. As a consequence of previous results, FA was suggested as a possible surrogate marker of lesion age in the very first hours of stroke [12] that might be used to infer time of symptom onset in patients with unknown time window in addition to other concepts like the DWI-FLAIR-mismatch. Based on our results we cannot support this idea. At least within the very first hours of stroke, FA does not provide helpful information about the age of the ischemic lesion.

Potentially diffusion kurtosis imaging (DKI), using a larger set of b-values data, based on a 2nd order approximation of the dependence of signal on b-values, will be more sensitive in identifying the age of the ischemic lesion, which needs to be studied in the future [18].

There are limitations to our study. We did not take into account possible anatomical contributions to acute FA changes as we did not include information on lesion location into our analysis. We also did not look at grey and white matter lesion compartments separately. Also FA may be related to factors as partial volume effects, different compartiments, crossing fibres or axon myelination. Further a mean FA value of all voxels within an ischemic stroke lesion of quite different lesion volume and location may not represent an adequate measure to be analyzed. We excluded contralateral stroke or severe leukoariosis but further influence of the contralateral VOI was not analysed. Finally, as our sample was restricted to patients studied within 8 hours of symptom onset, we cannot answer the question, whether a decreased FA allows for identification of acute ischemic stroke lesions beyond a later time point, e.g. >8, 12, or 24 hours of symptom onset.

## Conclusion

Within the first hours of acute cerebral ischemia, cytotoxic edema leads to decreased diffusivity along all axes with reduction of both isotropic and anisotropic components of diffusion. Increased FA in the ischemic lesion observed in the majority of patients within the very first hours of stroke results from a more pronounced decrease of the isotropic as compared to the anisotropic diffusion component and is not related to time from symptom onset. Thus, FA is not helpful as a surrogate marker of lesion age in the first hours of stroke.

## Supporting information

S1 Dataset Alegiani et al_data_set.pdf Anonymized data set of the study.
(PDF)
