# Supplementary material for: Comprehensive analysis of early fractional anisotropy changes in acute ischemic stroke
Source: PLoS One. 2017 Nov 30;12(11):e0188318. doi: 10.1371/journal.pone.0188318 (PMC5708650; doi:10.1371/journal.pone.0188318)
Supplement: S1 Dataset — Anonymized data set of the study. (PDF) [file pone.0188318.s001.pdf]

patient	parameter	gender (male=1, female=2)	age
9	FA	1	68
9	L1		
9	L2		
9	L3		
9	MD		
9	S0		
9	rad.diff.		
13	FA	2	43
13	L1		
13	L2		
13	L3		
13	MD		
13	S0		
13	rad.diff.		
29	FA	1	86
29	L1		
29	L2		
29	L3		
29	MD		
29	S0		
29	rad.diff.		
43	FA	1	66
43	L1		
43	L2		
43	L3		
43	MD		
43	S0		
43	rad.diff.		
44	FA	1	84
44	L1		
44	L2		
44	L3		
44	MD		
44	S0		
44	rad.diff.		
45	FA	1	57
45	L1		
45	L2		
45	L3		
45	MD		
45	S0		
45	rad.diff.		
48	FA	1	70
48	L1		
48	L2		
48	L3		
48	MD		
48	S0		
48	rad.diff.		

62	FA	1	80
62	L1		
62	L2		
62	L3		
62	MD		
62	S0		
62	rad.diff.		
74	FA	2	80
74	L1		
74	L2		
74	L3		
74	MD		
74	S0		
74	rad.diff.		
108	FA	2	36
108	L1		
108	L2		
108	L3		
108	MD		
108	S0		
108	rad.diff.		
109	FA	2	79
109	L1		
109	L2		
109	L3		
109	MD		
109	S0		
109	rad.diff.		
110	FA	1	55
110	L1		
110	L2		
110	L3		
110	MD		
110	S0		
110	rad.diff.		
140	FA	2	43
140	L1		
140	L2		
140	L3		
140	MD		
140	S0		
140	rad.diff.		
149	FA	2	35
149	L1		
149	L2		
149	L3		
149	MD		
149	S0		
149	rad.diff.		
153	FA	1	66

153	L1		
153	L2		
153	L3		
153	MD		
153	S0		
153	rad.diff.		
170	FA	1	74
170	L1		
170	L2		
170	L3		
170	MD		
170	S0		
170	rad.diff.		
171	FA	2	47
171	L1		
171	L2		
171	L3		
171	MD		
171	S0		
171	rad.diff.		
187	FA	2	60
187	L1		
187	L2		
187	L3		
187	MD		
187	S0		
187	rad.diff.		
190	FA	2	66
190	L1		
190	L2		
190	L3		
190	MD		
190	S0		
190	rad.diff.		
193	FA	1	74
193	L1		
193	L2		
193	L3		
193	MD		
193	S0		
193	rad.diff.		
194	FA	1	62
194	L1		
194	L2		
194	L3		
194	MD		
194	S0		
194	rad.diff.		
195	FA	2	74
195	L1		

195	L2		
195	L3		
195	MD		
195	S0		
195	rad.diff.		
198	FA	2	79
198	L1		
198	L2		
198	L3		
198	MD		
198	S0		
198	rad.diff.		
199	FA	2	77
199	L1		
199	L2		
199	L3		
199	MD		
199	S0		
199	rad.diff.		
236	FA	1	75
236	L1		
236	L2		
236	L3		
236	MD		
236	S0		
236	rad.diff.		
249	FA	1	67
249	L1		
249	L2		
249	L3		
249	MD		
249	S0		
249	rad.diff.		
254	FA	1	73
254	L1		
254	L2		
254	L3		
254	MD		
254	S0		
254	rad.diff.		
255	FA	1	72
255	L1		
255	L2		
255	L3		
255	MD		
255	S0		
255	rad.diff.		
904	FA	2	74
904	L1		
904	L2		

904	L3		
904	MD		
904	S0		
904	rad.diff.		
905	FA	2	51
905	L1		
905	L2		
905	L3		
905	MD		
905	S0		
905	rad.diff.		
907	FA	2	69
907	L1		
907	L2		
907	L3		
907	MD		
907	S0		
907	rad.diff.		
908	FA	1	44
908	L1		
908	L2		
908	L3		
908	MD		
908	S0		
908	rad.diff.		
910	FA	1	67
910	L1		
910	L2		
910	L3		
910	MD		
910	S0		
910	rad.diff.		
911	FA	1	71
911	L1		
911	L2		
911	L3		
911	MD		
911	S0		
911	rad.diff.		
912	FA	1	82
912	L1		
912	L2		
912	L3		
912	MD		
912	S0		
912	rad.diff.		
915	FA	1	77
915	L1		
915	L2		
915	L3		

915	MD		
915	S0		
915	rad.diff.		
916	FA	2	48
916	L1		
916	L2		
916	L3		
916	MD		
916	S0		
916	rad.diff.		
917	FA	1	78
917	L1		
917	L2		
917	L3		
917	MD		
917	S0		
917	rad.diff.		
918	FA	2	92
918	L1		
918	L2		
918	L3		
918	MD		
918	S0		
918	rad.diff.		
919	FA	1	62
919	L1		
919	L2		
919	L3		
919	MD		
919	S0		
919	rad.diff.		
922	FA	1	78
922	L1		
922	L2		
922	L3		
922	MD		
922	S0		
922	rad.diff.		
925	FA	2	60
925	L1		
925	L2		
925	L3		
925	MD		
925	S0		
925	rad.diff.		
927	FA	1	78
927	L1		
927	L2		
927	L3		
927	MD		

927	S0		
927	rad.diff.		
928	FA	1	68
928	L1		
928	L2		
928	L3		
928	MD		
928	S0		
928	rad.diff.		
929	FA	2	78
929	L1		
929	L2		
929	L3		
929	MD		
929	S0		
929	rad.diff.		
930	FA	2	83
930	L1		
930	L2		
930	L3		
930	MD		
930	S0		
930	rad.diff.		
931	FA	1	80
931	L1		
931	L2		
931	L3		
931	MD		
931	S0		
931	rad.diff.		
933	FA	1	61
933	L1		
933	L2		
933	L3		
933	MD		
933	S0		
933	rad.diff.		
934	FA	2	47
934	L1		
934	L2		
934	L3		
934	MD		
934	S0		
934	rad.diff.		
937	FA	2	65
937	L1		
937	L2		
937	L3		
937	MD		
937	S0		

937	rad.diff.		
938	FA	2	89
938	L1		
938	L2		
938	L3		
938	MD		
938	S0		
938	rad.diff.		
939	FA	1	71
939	L1		
939	L2		
939	L3		
939	MD		
939	S0		
939	rad.diff.		
940	FA	2	73
940	L1		
940	L2		
940	L3		
940	MD		
940	S0		
940	rad.diff.		
941	FA	2	60
941	L1		
941	L2		
941	L3		
941	MD		
941	S0		
941	rad.diff.		
942	FA	2	58
942	L1		
942	L2		
942	L3		
942	MD		
942	S0		
942	rad.diff.		
943	FA	2	84
943	L1		
943	L2		
943	L3		
943	MD		
943	S0		
943	rad.diff.		
946	FA	1	43
946	L1		
946	L2		
946	L3		
946	MD		
946	S0		
946	rad.diff.		

947	FA	2	78
947	L1		
947	L2		
947	L3		
947	MD		
947	S0		
947	rad.diff.		
949	FA	1	82
949	L1		
949	L2		
949	L3		
949	MD		
949	S0		
949	rad.diff.		
951	FA	2	95
951	L1		
951	L2		
951	L3		
951	MD		
951	S0		
951	rad.diff.		
952	FA	2	90
952	L1		
952	L2		
952	L3		
952	MD		
952	S0		
952	rad.diff.		
954	FA	1	65
954	L1		
954	L2		
954	L3		
954	MD		
954	S0		
954	rad.diff.		
956	FA	2	73
956	L1		
956	L2		
956	L3		
956	MD		
956	S0		
956	rad.diff.		

*Lesion size: 1= lacunar infarction, 2=< 1/3 of media territory, 3

time from stroke onset to MRI [h:mm:ss]	side of infarction (left=1, right=2)	lesion size*	NIHSS
6:43:00	1	3	8
6:43:00	1	3	
6:43:00	1	3	
6:43:00	1	3	
6:43:00	1	3	
6:43:00	1	3	
6:43:00	1	3	
2:31:00	1	2	6
2:31:00	1	2	
2:31:00	1	2	
2:31:00	1	2	
2:31:00	1	2	
2:31:00	1	2	
2:31:00	1	2	
1:26:00	1	4	12
1:26:00	1	4	
1:26:00	1	4	
1:26:00	1	4	
1:26:00	1	4	
1:26:00	1	4	
1:26:00	1	4	
4:36:00	1	2	10
4:36:00	1	2	
4:36:00	1	2	
4:36:00	1	2	
4:36:00	1	2	
4:36:00	1	2	
4:36:00	1	2	
2:32:00	2	2	4
2:32:00	2	2	
2:32:00	2	2	
2:32:00	2	2	
2:32:00	2	2	
2:32:00	2	2	
2:32:00	2	2	
3:01:00	1	1	10
3:01:00	1	1	
3:01:00	1	1	
3:01:00	1	1	
3:01:00	1	1	
3:01:00	1	1	
3:01:00	1	1	
2:22:00	1	3	7
2:22:00	1	3	
2:22:00	1	3	
2:22:00	1	3	
2:22:00	1	3	
2:22:00	1	3	
2:22:00	1	3	

2:09:00	1	4	17
2:09:00	1	4	
2:09:00	1	4	
2:09:00	1	4	
2:09:00	1	4	
2:09:00	1	4	
2:09:00	1	4	
2:03:00	2	2	16
2:03:00	2	2	
2:03:00	2	2	
2:03:00	2	2	
2:03:00	2	2	
2:03:00	2	2	
2:03:00	2	2	
3:10:00	2	2	1
3:10:00	2	2	
3:10:00	2	2	
3:10:00	2	2	
3:10:00	2	2	
3:10:00	2	2	
3:10:00	2	2	
4:30:00	2	2	17
4:30:00	2	2	
4:30:00	2	2	
4:30:00	2	2	
4:30:00	2	2	
4:30:00	2	2	
4:30:00	2	2	
2:41:00	1	1	6
2:41:00	1	1	
2:41:00	1	1	
2:41:00	1	1	
2:41:00	1	1	
2:41:00	1	1	
2:41:00	1	1	
1:54:00	2	2	5
1:54:00	2	2	
1:54:00	2	2	
1:54:00	2	2	
1:54:00	2	2	
1:54:00	2	2	
1:54:00	2	2	
5:46:00	1	2	4
5:46:00	1	2	
5:46:00	1	2	
5:46:00	1	2	
5:46:00	1	2	
5:46:00	1	2	
5:46:00	1	2	
1:25:00	1	3	18

1:25:00	1	3	
1:25:00	1	3	
1:25:00	1	3	
1:25:00	1	3	
1:25:00	1	3	
1:25:00	1	3	
4:44:00	1	3	14
4:44:00	1	3	
4:44:00	1	3	
4:44:00	1	3	
4:44:00	1	3	
4:44:00	1	3	
4:44:00	1	3	
5:21:00	2	3	11
5:21:00	2	3	
5:21:00	2	3	
5:21:00	2	3	
5:21:00	2	3	
5:21:00	2	3	
5:21:00	2	3	
1:55:00	1	2	2
1:55:00	1	2	
1:55:00	1	2	
1:55:00	1	2	
1:55:00	1	2	
1:55:00	1	2	
1:55:00	1	2	
5:44:00	2	2	4
5:44:00	2	2	
5:44:00	2	2	
5:44:00	2	2	
5:44:00	2	2	
5:44:00	2	2	
5:44:00	2	2	
1:11:00	1	4	22
1:11:00	1	4	
1:11:00	1	4	
1:11:00	1	4	
1:11:00	1	4	
1:11:00	1	4	
1:11:00	1	4	
1:46:00	2	1	6
1:46:00	2	1	
1:46:00	2	1	
1:46:00	2	1	
1:46:00	2	1	
1:46:00	2	1	
1:46:00	2	1	
1:14:00	2	3	4
1:14:00	2	3	

1:14:00	2	3	
1:14:00	2	3	
1:14:00	2	3	
1:14:00	2	3	
1:14:00	2	3	
2:17:00	2	4	20
2:17:00	2	4	
2:17:00	2	4	
2:17:00	2	4	
2:17:00	2	4	
2:17:00	2	4	
2:17:00	2	4	
2:52:00	1	2	21
2:52:00	1	2	
2:52:00	1	2	
2:52:00	1	2	
2:52:00	1	2	
2:52:00	1	2	
2:52:00	1	2	
2:01:00	1	2	6
2:01:00	1	2	
2:01:00	1	2	
2:01:00	1	2	
2:01:00	1	2	
2:01:00	1	2	
2:01:00	1	2	
3:08:00	2	4	8
3:08:00	2	4	
3:08:00	2	4	
3:08:00	2	4	
3:08:00	2	4	
3:08:00	2	4	
3:08:00	2	4	
2:38:00	1	4	22
2:38:00	1	4	
2:38:00	1	4	
2:38:00	1	4	
2:38:00	1	4	
2:38:00	1	4	
2:38:00	1	4	
1:02:00	1	3	20
1:02:00	1	3	
1:02:00	1	3	
1:02:00	1	3	
1:02:00	1	3	
1:02:00	1	3	
1:02:00	1	3	
1:22:00	1	2	3
1:22:00	1	2	
1:22:00	1	2	

1:22:00	1	2	
1:22:00	1	2	
1:22:00	1	2	
1:22:00	1	2	
0:44:00	1	2	3
0:44:00	1	2	
0:44:00	1	2	
0:44:00	1	2	
0:44:00	1	2	
0:44:00	1	2	
0:44:00	1	2	
1:42:00	2	2	18
1:42:00	2	2	
1:42:00	2	2	
1:42:00	2	2	
1:42:00	2	2	
1:42:00	2	2	
1:42:00	2	2	
2:23:00	2	2	4
2:23:00	2	2	
2:23:00	2	2	
2:23:00	2	2	
2:23:00	2	2	
2:23:00	2	2	
2:23:00	2	2	
0:21:00	1	4	19
0:21:00	1	4	
0:21:00	1	4	
0:21:00	1	4	
0:21:00	1	4	
0:21:00	1	4	
0:21:00	1	4	
4:09:00	1	2	9
4:09:00	1	2	
4:09:00	1	2	
4:09:00	1	2	
4:09:00	1	2	
4:09:00	1	2	
4:09:00	1	2	
4:06:00	1	2	4
4:06:00	1	2	
4:06:00	1	2	
4:06:00	1	2	
4:06:00	1	2	
4:06:00	1	2	
4:06:00	1	2	
1:47:00	1	1	12
1:47:00	1	1	
1:47:00	1	1	
1:47:00	1	1	

1:47:00	1	1	
1:47:00	1	1	
1:47:00	1	1	
3:14:00	1	4	20
3:14:00	1	4	
3:14:00	1	4	
3:14:00	1	4	
3:14:00	1	4	
3:14:00	1	4	
3:14:00	1	4	
3:45:00	1	1	5
3:45:00	1	1	
3:45:00	1	1	
3:45:00	1	1	
3:45:00	1	1	
3:45:00	1	1	
3:45:00	1	1	
2:41:00	2	3	7
2:41:00	2	3	
2:41:00	2	3	
2:41:00	2	3	
2:41:00	2	3	
2:41:00	2	3	
2:41:00	2	3	
1:17:00	1	2	8
1:17:00	1	2	
1:17:00	1	2	
1:17:00	1	2	
1:17:00	1	2	
1:17:00	1	2	
1:17:00	1	2	
1:26:00	1	2	3
1:26:00	1	2	
1:26:00	1	2	
1:26:00	1	2	
1:26:00	1	2	
1:26:00	1	2	
1:26:00	1	2	
3:15:00	1	1	4
3:15:00	1	1	
3:15:00	1	1	
3:15:00	1	1	
3:15:00	1	1	
3:15:00	1	1	
3:15:00	1	1	
2:00:00	1	1	5
2:00:00	1	1	
2:00:00	1	1	
2:00:00	1	1	
2:00:00	1	1	

2:00:00	1	1	
2:00:00	1	1	
3:32:00	2	3	16
3:32:00	2	3	
3:32:00	2	3	
3:32:00	2	3	
3:32:00	2	3	
3:32:00	2	3	
3:32:00	2	3	
1:49:00	1	2	26
1:49:00	1	2	
1:49:00	1	2	
1:49:00	1	2	
1:49:00	1	2	
1:49:00	1	2	
1:49:00	1	2	
7:25:00	2	4	27
7:25:00	2	4	
7:25:00	2	4	
7:25:00	2	4	
7:25:00	2	4	
7:25:00	2	4	
7:25:00	2	4	
1:02:00	1	1	4
1:02:00	1	1	
1:02:00	1	1	
1:02:00	1	1	
1:02:00	1	1	
1:02:00	1	1	
1:02:00	1	1	
0:52:00	2	2	9
0:52:00	2	2	
0:52:00	2	2	
0:52:00	2	2	
0:52:00	2	2	
0:52:00	2	2	
0:52:00	2	2	
1:34:00	2	3	11
1:34:00	2	3	
1:34:00	2	3	
1:34:00	2	3	
1:34:00	2	3	
1:34:00	2	3	
1:34:00	2	3	
1:31:00	2	1	7
1:31:00	2	1	
1:31:00	2	1	
1:31:00	2	1	
1:31:00	2	1	
1:31:00	2	1	

1:31:00	2	1	
2:55:00	2	3	15
2:55:00	2	3	
2:55:00	2	3	
2:55:00	2	3	
2:55:00	2	3	
2:55:00	2	3	
2:55:00	2	3	
2:48:00	2	2	3
2:48:00	2	2	
2:48:00	2	2	
2:48:00	2	2	
2:48:00	2	2	
2:48:00	2	2	
2:48:00	2	2	
1:12:00	2	1	6
1:12:00	2	1	
1:12:00	2	1	
1:12:00	2	1	
1:12:00	2	1	
1:12:00	2	1	
1:12:00	2	1	
2:43:00	1	2	10
2:43:00	1	2	
2:43:00	1	2	
2:43:00	1	2	
2:43:00	1	2	
2:43:00	1	2	
2:43:00	1	2	
1:15:00	2	2	15
1:15:00	2	2	
1:15:00	2	2	
1:15:00	2	2	
1:15:00	2	2	
1:15:00	2	2	
1:15:00	2	2	
2:03:00	1	1	9
2:03:00	1	1	
2:03:00	1	1	
2:03:00	1	1	
2:03:00	1	1	
2:03:00	1	1	
2:03:00	1	1	
0:56:00	1	4	9
0:56:00	1	4	
0:56:00	1	4	
0:56:00	1	4	
0:56:00	1	4	
0:56:00	1	4	
0:56:00	1	4	

3:58:00	2	1	6
3:58:00	2	1	
3:58:00	2	1	
3:58:00	2	1	
3:58:00	2	1	
3:58:00	2	1	
3:58:00	2	1	
1:24:00	2	2	12
1:24:00	2	2	
1:24:00	2	2	
1:24:00	2	2	
1:24:00	2	2	
1:24:00	2	2	
1:24:00	2	2	
1:00:00	1	2	17
1:00:00	1	2	
1:00:00	1	2	
1:00:00	1	2	
1:00:00	1	2	
1:00:00	1	2	
1:00:00	1	2	
3:06:00	2	1	4
3:06:00	2	1	
3:06:00	2	1	
3:06:00	2	1	
3:06:00	2	1	
3:06:00	2	1	
3:06:00	2	1	
1:29:00	1	3	16
1:29:00	1	3	
1:29:00	1	3	
1:29:00	1	3	
1:29:00	1	3	
1:29:00	1	3	
1:29:00	1	3	
0:16:00	2	2	10
0:16:00	2	2	
0:16:00	2	2	
0:16:00	2	2	
0:16:00	2	2	
0:16:00	2	2	
0:16:00	2	2	

3=1/3 to 2/3 of media territory, 4=> 2/3 of media territory.

systolic blood pressure [mmHg]	blood glucose [mg/dl]	hematocrit [%]
126	111,724	43
140	99,0199	41
120		41
167	100,7318	42
130	98,5694	
140	99,11	33
120	124,338	

140	158,576	
153	148,1244	
107	114,2468	36
202	134,0688	45
125	119,4726	45
119	123,437	
140	85,7752	35
135	244,3512	43

150	150,467	46
130	108,12	
179	100,1912	39
120	102,3536	46
150	126,14	
218	100,5516	46
145	126,14	

196	97,8486	44
160	97,308	37
126	108,12	37
145	108,12	
171	145,7818	46
130	108,12	
174	110	40,3

160	109	44,2
142	131	33,3
146	88	43,1
	100	45,3
140	92	26,6
190	113	46,1
117	111	37,5

188	144	39,7
201	224	37,6
182	115	34,2
134	109	38,9
157	81	36,8
181	116	39,9
230	86	48

96	126	36,8
128	229	30,3
159	126	36
192	105	44,4
158	193	41,9
139	114	42,2
163	117	33,5

155	129	41,6
133	110	38,8
155	138	35,3
147	143	36,4
136	101	44,2
161	193	25,8
116	101	26,5

178	141	42,7
165		31,2
127	89	30,3
155	136	41,5
166	174	40,2
159		34

leucocyte count [*109/l]	plateled count [*109/l]	value_infarct lesion	value_contralateral
10,43	257	0,294629	0,313145
		0,000823	0,001131
		0,000604	0,0008
		0,000455	0,00061
		0,000627	0,000847
		244,06558	220,091933
		0,0005295	0,000705
7,9	248	0,250608	0,263807
		0,000704	0,000957
		0,000536	0,000722
		0,000432	0,000579
		0,000557	0,000753
		255,060054	213,224804
		0,000484	0,0006505
7,7	311	0,2163	0,235733
		0,000801	0,001076
		0,000628	0,000846
		0,000519	0,000686
		0,000649	0,000869
		312,684799	301,795182
		0,0005735	0,000766
6	234	0,274944	0,246542
		0,000699	0,000977
		0,000533	0,000753
		0,000413	0,000612
		0,000548	0,000781
		189,197535	176,52011
		0,000473	0,0006825
7,38	180	0,263807	0,292291
		0,000873	0,001055
		0,000671	0,000763
		0,000529	0,000613
		0,000691	0,00081
		227,340376	219,060809
		0,0006	0,000688
7	150	0,501261	0,514882
		0,00091	0,001119
		0,000467	0,000567
		0,00031	0,000386
		0,000562	0,00069
		203,326425	194,989337
		0,0003885	0,0004765
6,73	160	0,337311	0,320901
		0,000673	0,001048
		0,000469	0,000742
		0,000342	0,000571
		0,000495	0,000787
		233,847921	221,989218
		0,0004055	0,0006565

12,8	235	0,261846	0,2444
		0,000719	0,001053
		0,000561	0,000817
		0,000449	0,000657
		0,000576	0,000842
		309,617613	280,445917
		0,000505	0,000737
10,9	247	0,352337	0,384951
		0,000818	0,000977
		0,000582	0,000691
		0,000393	0,00043
		0,000598	0,000699
		234,349493	213,935263
		0,0004875	0,0005605
10,7	279	0,189913	0,128682
		0,000862	0,000924
		0,000686	0,000798
		0,000596	0,000722
		0,000715	0,000815
		273,547042	274,285562
		0,000641	0,00076
20,61	582	0,27723	0,300491
		0,000815	0,001124
		0,000581	0,00078
		0,000471	0,000629
		0,000622	0,000845
		299,063049	270,266553
		0,000526	0,0007045
12,3	304	0,168662	0,201205
		0,001129	0,001168
		0,000955	0,000958
		0,000814	0,000789
		0,000966	0,000972
		366,727179	370,19948
		0,0008845	0,0008735
17	335	0,296199	0,291927
		0,000719	0,000875
		0,000517	0,000643
		0,000391	0,000491
		0,000543	0,00067
		262,318924	246,656674
		0,000454	0,000567
12	709	0,262756	0,215131
		0,000734	0,000956
		0,000555	0,000775
		0,000444	0,000619
		0,000575	0,000782
		196,964268	192,875134
		0,0004995	0,000697
9,53	248	0,17652	0,176699

		0,000681	0,000963
		0,000572	0,000798
		0,000502	0,000698
		0,000585	0,00082
		251,184179	271,185494
		0,000537	0,000748
8,6	271	0,198011	0,190072
		0,000711	0,000983
		0,000578	0,000804
		0,000496	0,000692
		0,000595	0,000827
		245,095501	254,264179
		0,000537	0,000748
6	230	0,344165	0,336033
		0,000816	0,000951
		0,000548	0,000645
		0,000407	0,00048
		0,000591	0,000692
		259,573841	235,426039
		0,0004775	0,0005625
4,37	419	0,297351	0,306311
		0,000799	0,000947
		0,000579	0,000657
		0,000438	0,000522
		0,000605	0,000709
		272,528832	273,737046
		0,0005085	0,0005895
10,29	254	0,36994	0,34073
		0,000744	0,000929
		0,0005	0,000635
		0,000349	0,000461
		0,000531	0,000675
		265,280307	222,220254
		0,0004245	0,000548
7	200	0,246374	0,257682
		0,000741	0,001101
		0,00056	0,000818
		0,00046	0,000673
		0,000587	0,000864
		267,829287	274,386531
		0,00051	0,0007455
9,63	292	0,401974	0,353224
		0,000952	0,001061
		0,000554	0,000661
		0,000433	0,000538
		0,000646	0,000754
		254,718643	247,506773
		0,0004935	0,0005995
7	190	0,232907	0,24617
		0,000862	0,000927

		0,000648	0,000702
		0,00054	0,000563
		0,000683	0,000731
		252,298064	222,882108
		0,000594	0,0006325
13,4	237	0,291549	0,241652
		0,000726	0,001036
		0,000532	0,000804
		0,000408	0,000651
		0,000556	0,00083
		282,268174	236,035854
		0,00047	0,0007275
20,4	400	0,251883	0,243722
		0,000731	0,000953
		0,000575	0,000735
		0,000448	0,000598
		0,000585	0,000762
		263,334541	253,698483
		0,0005115	0,0006665
6,45	224	0,231142	0,238287
		0,000867	0,001012
		0,000673	0,000781
		0,000562	0,000646
		0,000701	0,000813
		272,741388	262,247969
		0,0006175	0,0007135
7	240	0,211208	0,204646
		0,000726	0,000977
		0,000575	0,000791
		0,000488	0,000669
		0,000596	0,000812
		330,85554	278,02464
		0,0005315	0,00073
7,6	152	0,19998	0,202766
		0,000692	0,000967
		0,000565	0,000774
		0,000477	0,000662
		0,000578	0,000801
		267,341497	235,273859
		0,000521	0,000718
7	240	0,356437	0,332047
		0,000828	0,001048
		0,000551	0,000688
		0,000414	0,000553
		0,000598	0,000763
		266,342505	264,848265
		0,0004825	0,0006205
10,8	318	0,238896	0,273284
		0,000768	0,001097
		0,000608	0,000822

		0,000485	0,000653
		0,00062	0,000857
		343,203706	322,746246
		0,0005465	0,0007375
6,3	393	0,260806	0,222696
		0,000648	0,000994
		0,000504	0,000794
		0,000392	0,000659
		0,000515	0,000816
		286,881801	277,475618
		0,000448	0,0007265
2,6	221	0,427614	0,413645
		0,000791	0,001054
		0,00048	0,000648
		0,000324	0,000461
		0,000532	0,000721
		240,931922	247,262396
		0,000402	0,0005545
8,2	200	0,247664	0,206532
		0,000807	0,001034
		0,000624	0,000846
		0,000506	0,000707
		0,000646	0,000862
		327,405578	330,290457
		0,000565	0,0007765
9	279	0,432913	0,320186
		0,000948	0,001156
		0,000666	0,000871
		0,000367	0,000599
		0,000659	0,000876
		240,297456	248,797597
		0,0005165	0,000735
5,7	229	0,40387	0,369346
		0,000793	0,001122
		0,000556	0,0008
		0,000352	0,000533
		0,000567	0,000818
		237,143529	325,624825
		0,000454	0,0006665
7,8	273	0,33116	0,305222
		0,000968	0,001162
		0,0007	0,000846
		0,000493	0,000623
		0,00072	0,000877
		282,72468	292,074792
		0,0005965	0,0007345
4,6	212	0,224719	0,184836
		0,000731	0,001118
		0,000576	0,000947
		0,000473	0,000785

		0,000593	0,00095
		346,385154	366,444624
		0,0005245	0,000866
8,5	272	0,330684	0,296544
		0,000649	0,000972
		0,000468	0,000715
		0,000344	0,000555
		0,000487	0,000747
		265,718906	233,665393
		0,000406	0,000635
5,5	179	0,329809	0,321054
		0,00067	0,001448
		0,000511	0,00103
		0,000353	0,000753
		0,000512	0,001077
		266,601839	275,359051
		0,000432	0,0008915
5,9	160	0,267956	0,245997
		0,000785	0,001139
		0,000614	0,000884
		0,000475	0,000719
		0,000624	0,000913
		335,370187	255,181066
		0,0005445	0,0008015
8,2	302	0,38309	0,305515
		0,000814	0,001041
		0,000521	0,000744
		0,000386	0,000566
		0,000573	0,000784
		261,844615	263,889054
		0,0004535	0,000655
6,7	217	0,299187	0,325401
		0,000954	0,001326
		0,000682	0,000895
		0,000537	0,000703
		0,000724	0,000975
		395,955529	387,932463
		0,0006095	0,000799
10,8	300	0,287912	0,330015
		0,000693	0,001048
		0,000526	0,000747
		0,000385	0,000535
		0,000535	0,000777
		303,727334	263,898091
		0,0004555	0,000641
10,8	256	0,358265	0,330551
		0,000575	0,001116
		0,000444	0,000814
		0,00028	0,00058
		0,000419	0,000837

		258,826844	313,366017
		0,000362	0,000697
11,6	108	0,3329	0,27985
		0,000641	0,001051
		0,000462	0,000787
		0,000331	0,00062
		0,000478	0,000819
		319,23311	282,602814
		0,0003965	0,0007035
7,5	480	0,515734	0,50608
		0,00075	0,001225
		0,000385	0,000613
		0,000256	0,000466
		0,000458	0,000763
		248,124054	260,712948
		0,0003205	0,0005395
12,6	280	0,237925	0,223154
		0,000728	0,001107
		0,000574	0,000895
		0,000462	0,000729
		0,000588	0,00091
		382,614852	322,227076
		0,000518	0,000812
9,4	386	0,329447	0,28651
		0,00084	0,001033
		0,000603	0,000779
		0,00043	0,000581
		0,000625	0,000798
		319,623989	335,006328
		0,0005165	0,00068
6,8	196	0,394642	0,335487
		0,000851	0,001106
		0,000545	0,000754
		0,000383	0,000569
		0,000593	0,00081
		262,432027	261,78882
		0,000464	0,0006615
9	266	0,404568	0,358557
		0,00069	0,001004
		0,000438	0,000661
		0,000306	0,000501
		0,000477	0,000721
		244,3606	239,301338
		0,000372	0,000581
5,2	249	0,432913	0,419961
		0,000982	0,001168
		0,000566	0,000679
		0,000415	0,000513
		0,000654	0,000787
		295,949202	279,839481

		0,0004905	0,000596
9,9	380	0,362933	0,361566
		0,0007	0,001166
		0,000459	0,000747
		0,000347	0,000582
		0,000502	0,000831
		321,684274	290,374069
		0,000403	0,0006645
7,6	209	0,253916	0,236714
		0,000696	0,001048
		0,000538	0,000857
		0,000428	0,000673
		0,000554	0,000857
		388,98628	325,66154
		0,000483	0,000765
6,7	242	0,56046	0,519131
		0,001033	0,001102
		0,000479	0,000529
		0,000305	0,000361
		0,000605	0,000664
		284,391777	261,288797
		0,000392	0,000445
13,7	304	0,417364	0,222052
		0,000491	0,000995
		0,000363	0,000805
		0,000226	0,000658
		0,000357	0,000819
		345,916358	293,488596
		0,0002945	0,0007315
5,9	190	0,381382	0,383289
		0,000692	0,000955
		0,000458	0,000634
		0,000312	0,000431
		0,000487	0,000674
		253,562259	237,569835
		0,000385	0,0005325
9,2	233	0,282291	0,269197
		0,000862	0,001098
		0,000634	0,000797
		0,000501	0,000662
		0,000666	0,000852
		284,436446	272,999781
		0,0005675	0,0007295
8,7	433	0,336035	0,2686
		0,000827	0,001128
		0,000599	0,000861
		0,000421	0,000673
		0,000615	0,000888
		284,336366	283,46919
		0,00051	0,000767

8,9	247	0,420097	0,360669
		0,000782	0,001059
		0,000534	0,000698
		0,000349	0,00054
		0,000554	0,000765
		299,058362	297,337552
		0,0004415	0,000619
14,1	133	0,342969	0,276746
		0,000771	0,001017
		0,000554	0,000772
		0,000409	0,000613
		0,000578	0,0008
		296,069662	305,073956
		0,0004815	0,0006925
5,1	203	0,159896	0,199371
		0,001027	0,001227
		0,00089	0,000989
		0,00076	0,000835
		0,000893	0,001017
		401,280192	341,36939
		0,000825	0,000912
7,3	267	0,366799	0,304809
		0,000876	0,001092
		0,000567	0,000789
		0,000424	0,00061
		0,000623	0,00083
		313,234104	325,534096
		0,0004955	0,0006995
10,7	249	0,349676	0,308454
		0,000702	0,001019
		0,000479	0,000734
		0,000347	0,000565
		0,000509	0,000772
		265,169408	264,742139
		0,000413	0,0006495
5,3	274	0,276278	0,191156
		0,000714	0,001186
		0,000539	0,000993
		0,000413	0,000827
		0,000555	0,001002
		406,096516	378,122242
		0,000476	0,00091
